# ORTI: An Open-Access Repository of Transcriptional Interactions for Interrogating Mammalian Gene Expression Data

**DOI:** 10.1371/journal.pone.0164535

**Published:** 2016-10-10

**Authors:** Fatemeh Vafaee, James R. Krycer, Xiuquan Ma, Timur Burykin, David E. James, Zdenka Kuncic

**Affiliations:** 1 Charles Perkins Centre, The University of Sydney, Sydney, NSW, Australia; 2 School of Mathematics and Statistics, The University of Sydney, Sydney, NSW, Australia; 3 School of Life and Environmental Sciences, The University of Sydney, Sydney, NSW, Australia; 4 Diabetes and Metabolism Division, Garvan Institute of Medical Research, Darlinghurst, Sydney, NSW, Australia; 5 Sydney Medical School, The University of Sydney, Sydney, NSW, Australia; 6 School of Physics, The University of Sydney, Sydney, NSW, Australia; Friedrich-Alexander-Universitat Erlangen-Nurnberg, GERMANY

## Abstract

Transcription factors (TFs) play a fundamental role in coordinating biological processes in response to stimuli. Consequently, we often seek to determine the key TFs and their regulated target genes (TGs) amidst gene expression data. This requires a knowledge-base of TF-TG interactions, which would enable us to determine the topology of the transcriptional network and predict novel regulatory interactions. To address this, we generated an Open-access Repository of Transcriptional Interactions, ORTI, by integrating available TF-TG interaction databases. These databases rely on different types of experimental evidence, including low-throughput assays, high-throughput screens, and bioinformatics predictions. We have subsequently categorised TF-TG interactions in ORTI according to the quality of this evidence. To demonstrate its capabilities, we applied ORTI to gene expression data and identified modulated TFs using an enrichment analysis. Combining this with pairwise TF-TG interactions enabled us to visualise temporal regulation of a transcriptional network. Additionally, ORTI enables the prediction of novel TF-TG interactions, based on how well candidate genes co-express with known TGs of the target TF. By filtering out known TF-TG interactions that are unlikely to occur within the experimental context, this analysis predicts context-specific TF-TG interactions. We show that this can be applied to experimental designs of varying complexities. In conclusion, ORTI is a rich and publicly available database of experimentally validated mammalian transcriptional interactions which is accompanied with tools that can identify and predict transcriptional interactions, serving as a useful resource for unravelling the topology of transcriptional networks.

## Introduction

The ever increasing popularity in ‘omics’ technologies has led to an explosion of data on individual molecules, from which we aim to infer their relationships. In the case of gene expression data, we often seek to determine the transcriptional regulators driving their expression, not only for mechanistic insight but also to better understand how biological processes are coordinated in response to stimuli.

There are several approaches for interrogating expression data *in silico*. For instance, one can search for motifs in gene promoters [1, 2]. This assumes that each transcription factor (TF) recognises unique promoter motifs. Although many such motifs are known, motif-searching within DNA sequence data from higher organisms requires tailored statistical analysis for each TF [3] and has been limited by false predictions [2]—for instance, there would be a high occurrence of a small 6–10 bp motif across the genome, but relatively few sites would be of functional importance. An alternative approach involves using prior gene expression studies to derive gene signatures, a common set of expression changes that occur in response to a perturbation such as knock-down of a TF [4]. Detecting these in the candidate dataset implies a contribution from that TF. Whilst this is suitable for detecting TFs represented in a dataset, this does not enable the prediction of direct relationships between TFs and their target genes (TGs) because the gene signatures do not distinguish between primary effects (e.g., TF binding to the TG promoter) and secondary effects (e.g., TF modulating the expression of a direct regulator of the TG). Overall, TFs have overlapping effects on TGs, whereby the activity of multiple TFs can influence the expression of a single gene. To overcome this, a database of experimentally-validated, direct TF-TG interactions is required. Such a knowledge base has been used, for instance, to predict regulatory relationships in yeast microarray data, and it has been shown that high quality, comprehensive and validated knowledge bases can significantly improve the discovery of TF-TG interactions from high throughput gene expression data [5].

There are numerous mammalian TF-TG databases available online [1, 2, 4, 6–10]. However, some are not publicly available (e.g., TRANSFAC, [1]) or are not regularly updated. Other databases do not distinguish between interactions based on the reliability of high- versus low-throughput experimental evidence. This has motivated us to develop a new publicly-available database, the Open-access Repository of Transcriptional Interactions (ORTI), which overcomes these limitations. Compiling various available databases, ORTI contains interactions derived from a range of experimental conditions, including reliable, low-throughput (LTP) experiments as well as broader, high-throughput (HTP) experiments. We apply this database to microarray expression data to reveal transcriptional interactions in gene expression data, identifying key TFs driving the expression changes and combining pairwise TF-TG interactions to visualise the topology of a transcriptional network. We also used ORTI to predict novel transcriptional interactions, using known TF-TG interactions that occur within the experimental context. Overall, we demonstrate that it will serve as a useful tool for elucidating the complex, nonlinear nature of transcriptional networks.

## Results and Discussion

### Construction of the ORTI database

**Fig 1:**
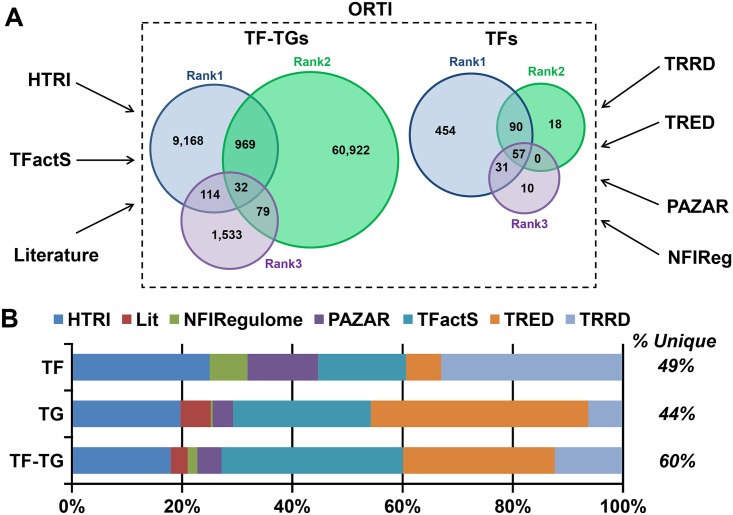
The construction of the ORTI database. Several publicly-available databases and literature references were merged to generate a collection of TF-TG interactions, classified as Rank 1, 2 or 3 according the reliability of the experimental validation. **A)** ORTI composition and Venn diagrams of the spread of Ranks over TFs and TF-TG interactions. **B)** The percentages of ORTI Rank 1 TFs, TGs, and TF-TGs which are unique to a single database and the contribution of each database.

The ORTI database was constructed by merging together several publicly-available databases and literature references to generate a collection of TF-TG interactions (Fig 1A). We considered TF and TG from mammalian model systems. Since the evidence for these interactions varies in quality, we have ranked the evidence according to experimental reliability: Rank 1, for LTP techniques such as electrophoretic mobility shift assays and promoter-based reporter assays, which are generally considered reliable methods for demonstrating that a TF binds to the TG’s promoter to regulate its expression; Rank 2, for HTP techniques such as chromatin immunoprecipitation coupled with sequencing (ChIP-Seq), which are informative but more susceptible to false positives compared to Rank 1 techniques; and Rank 3, for indirect evidence, including motif-based predictions and differential expression. This is detailed in S2 File.

Together, ORTI encompasses 688 TFs and 72,738 factor-gene interactions, out of which 10,370 regulations are Rank 1 (Table 1). Rank 2 data are naturally more abundant than Rank 1, but Rank 1 data still makes a relatively significant contribution to the database. Within the Rank 1 interactions, roughly half are unique to a single database (Fig 1B). Furthermore, although HTRI and TRRD together represent half of the TFs, TRED and TFactS contribute the majority of the TGs (Fig 1B). Thus, no single database dominates the ORTI. The flat-file for the database is available online (http://orti.sydney.edu.au). This online interface enables users to search for TF and/or TG names and aliases, providing suggestions for queries based on database content. Searches can be refined based on species and information source, and are sortable by rank. Results can be exported and multiple entries can be searched simultaneously, facilitating batch searching. Lastly, users are also able to submit new entries, which will be subject to manual curation prior to incorporation into the database.

**Table 1:** Number of TFs, TGs, and TF-TG interactions across all databases with no ranking constraint (all interactions) or when only LTP verified interactions (Rank 1) are included.

Database		# of TFs	# of TGs	# of TF-TG pairs
**ORTI**	LTP-only	632	3468	10,283
	All	660	20,146	72,817
**HTRI**	LTP-only	278	998	1,771
	All	284	18,298	51,872
**NFI-Reg**	LTP-only	49	54	152
	All	59	59	200
**PAZAR**	LTP-only	229	578	1,051
	All	250	4,868	8,449
**TFactS**	LTP-only	345	2,167	5,924
	All	345	2,617	6,727
**TRED**	LTP-only	144	2,078	4,724
	All	154	3,624	9,323
**TRRD**	LTP-only	321	607	1,296
	All	322	609	1,305

Overall, we have incorporated a rich variety of data sources to generate the largest publicly available database of TF-TG interactions to date (Table 1). Despite this, it is far from complete in terms of its coverage of the mammalian transcriptional interactome, given there are currently thousands of annotated TFs in the human genome [11, 12]. We intend to periodically update ORTI with experimentally validated factor-gene interactions. Furthermore, we encourage other researchers to submit any newly-discovered TF-TG interactions via the web interface. In addition to providing a repository for TF-TG interactions, we envisage that ORTI could be used to elucidate the transcriptional topology underlying gene expression data. We sought to achieve this via two applications, as discussed in the following sections. Both applications are developed in R and MATLAB. The fully commented source codes are available to download from the ORTI web interface.

### Identification of modulated TFs in different biological contexts

For each TF in ORTI, we considered TGs from any mammalian context, irrespective of cell type or species. This is justified by the conservation of gene regulation between species [4]. Our TF-TG interactions were also sourced from a range of experimental conditions—in light of this diversity, could ORTI be used to identify TFs that changed under specific biological conditions? To address this, we used our Application 1 algorithm: for each TF, we assessed whether its TGs from the ORTI database were over-represented within the genes differentially regulated in the expression data—e.g., |log_2_(fold change)| > 1.

Microarray studies were retrieved from NCBI GEO DataSets, using the keywords “expression profiling by array” [dataset] and “transcription factor”. We initially considered studies where a single TF had been modulated, such as by overexpression or knockdown, in either human or mouse cells [13–16]. Using ORTI, we could identify the modulated TF by a significantly enriched association of the TF within the differentially expressed genes, with *p*-value < 0.01 (Table 2), with the exception of PDX1 due to limited overlap between TGs and differentially regulated genes. The differentially expressed genes in these cases were obtained from the original papers. Next, we considered a study where a global stress was applied, in this case the induction of adipogenesis [17]. In this case, the raw data was acquired, and differentially expressed genes were identified according to the workflow depicted in Fig A of S3 File. By considering the early time-points (0-8h), we could detect known major regulators of adipogenesis [18] (Table 2). Notably, ORTI outperformed the other TF-TG interaction databases (Table 2)–in several instances, the other databases either did not contain the TF of interest or did not have sufficient TGs for that TF to enable a statistically significant enrichment. This was similarly found when just the Rank 1 data were considered (Table A of S3 File). This demonstrates that ORTI can be used to identify key TFs in different biological settings.

**Table 2:** ORTI outperforms other databases in identifying modulated TFs in gene expression data. TF enrichment analysis was performed on microarray data where a single TF was modified (AR [14], SREBF1 [16], PDX1 [13], E2F [15]), or for selected TFs in early adipogenesis [17]; *p*-values and Bonferroni corrected *p*-values are shown. ‘—‘ indicates that the TF was not found in the database. The parameters of the conducted hypergeometric tests are provided in Table B of S3 File.

Databases		Single TF modulated				Biological process			
		AR	SREBF1	E2F1	PDX1	CEBPA	CEBPB	CEBPD	PPARG
**ORTI**	*p*-value	0	1.42E-20	7.51E-16	5.77E-04	1.51E-20	9.19E-17	1.13E-10	2.41E-14
	Adj *p*-value	0	9.36E-18	4.96E-13	3.81E-01	9.94E-18	6.06E-14	7.47E-08	1.59E-11
**HTRI**	*p*-value	0	1	3.62E-03	1.66E-03	0.0115	6.60E-04	8.59E-03	0.168
	Adj *p*-value	0	1	1	0.4707	1	0.1874	1	1
**NFI-Reg**	*p*-value	—	—	—	—	—	—	0.055	—
	Adj *p*-value	—	—	—	—	—	—	1	—
**PAZAR**	*p*-value	1	—	0.0351	4.52E-03	0.539	0.279	1	—
	Adj *p*-value	1	—	1	1	1	1	1	—
**TFactS**	*p*-value	4.82E-07	9.99E-13	1.78E-04	0.0177	1.10E-05	0.0012	0.0313	4.09E-05
	Adj *p*-value	1.69E-04	3.45E-10	6.14E-02	1	3.79E-03	0.412	1	0.0141
**TRED**	*p*-value	1.04E-12	—	4.19E-03	—	5.87E-11	6.29E-08	6.77E-06	9.77E-08
	Adj *p*-value	1.60E-10	—	0.646	—	9.03E-09	9.69E-06	1.04E-03	1.51E-05
**TRRD**	*p*-value	0.0483	5.08E-04	1	1	0.0382	0.0382	0.122	0.479
	Adj *p*-value	1	0.1640	1	1	1	1	1	1

**Fig 2:**
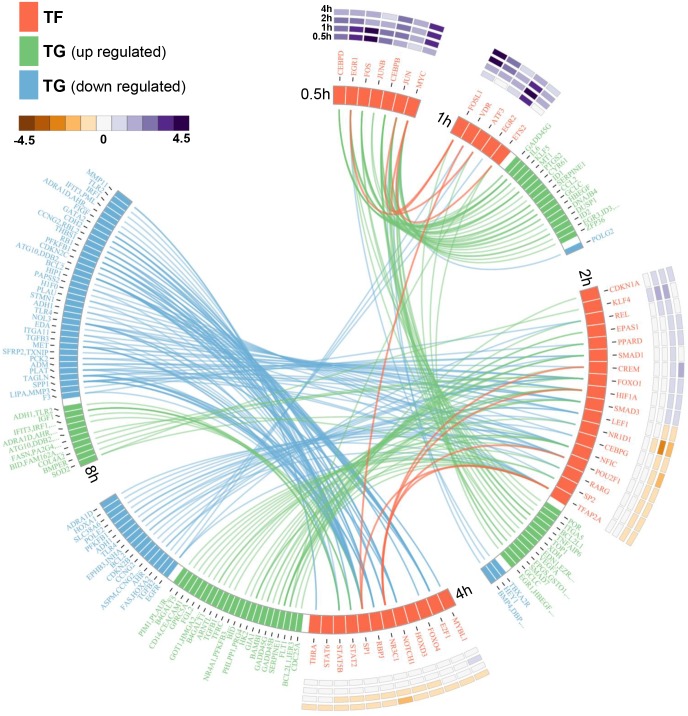
The transcriptional network during early adipogenesis. Using the adipogenesis time-course expression data [17], we identified differentially expressed genes (fold-change > 1.5, *p*-value < 0.05). Next, we combined differentially expressed genes from adjacent time-points (1h and 2h, 2h and 4h, etc), applying Application I to identify enriched TFs (*p*-value < 0.05). Combining two timepoints at a time provided sufficient resolution to observe rapid changes in TF activity. Enriched TFs that were differentially-expressed at the previous timepoint (e.g. at 0.5h, if enriched at 1-2h) were considered to be part of the transcriptional cascade. ORTI was used to identify the pairwise interactions that were subsequently used to construct the interaction network. This has been visualised using Circos [19]. The heatmap adjacent to each TF reflects their expression pattern over the time-course.

Next, we used the adipogenesis time-course data [17] to elucidate temporal changes in transcriptional network during early adipogenesis. This biological process is driven by transcriptional cascades, whereby one TF regulates the expression of another TF, influencing other TFs downstream [18]. To reveal these interactions, we first used Application 1 to determine when TFs were enriched (*p-*value<0.05) based on whether TGs were differentially expressed (*p*-value <0.05, and fold-change > 1.5) over time. These enriched TFs are putative regulators of adipogenesis. Next, we filtered for TFs that were themselves differentially expressed and thus likely to participate in a transcriptional cascade. Using ORTI, we then connected TFs together and to their TGs, visualising this using Circos [19]. This revealed a series of regulatory events over time (Fig 2), demonstrating that transcriptional cascades can be identified in an unbiased fashion using ORTI.

### Prediction of novel TF-TG interactions within specific biological contexts

**Fig 3:**
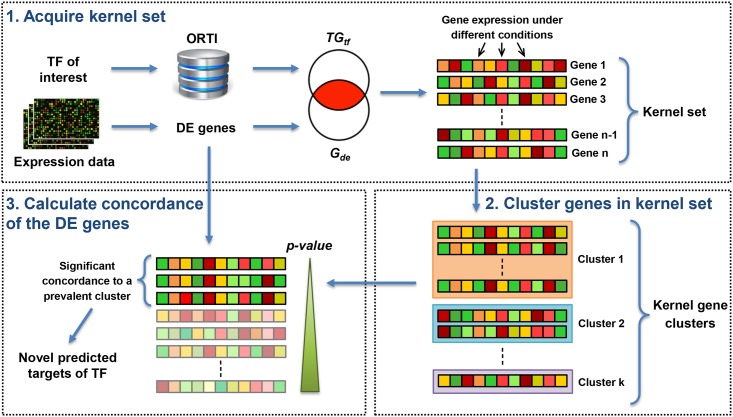
The pipe-line for Application II, the prediction of novel TF-TG interactions. Details are provided in the Materials and Methods. Abbreviations: TF, transcription factor; DE, differentially-expressed; TG_tf_, Rank 1 target genes connected to the TF of interest within the ORTI database; G_de_, subset of DE genes; KSC, kernel set concordance. Figure design partially adapted from [38].

**Fig 4:**
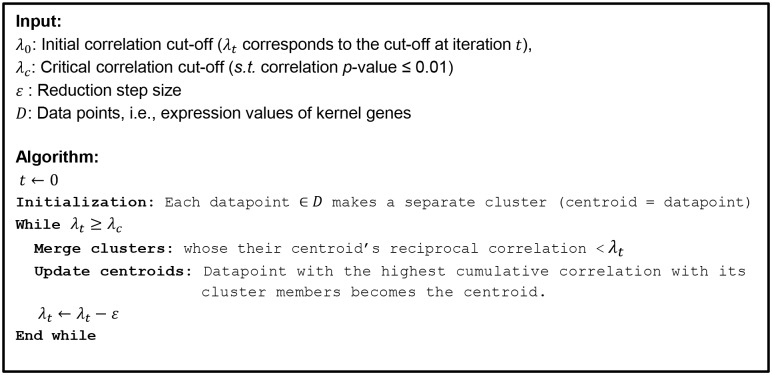
Clustering component for Application II. The proposed clustering algorithm used to identify prevalent TG expression patterns and singletons within the kernel set.

We also tested whether ORTI can be used to identify novel TF-TG interactions from gene expression data. This relies on the premise that a TF co-regulates multiple TGs within a biological context. For each TF, we use ORTI to identify known TGs that are differentially-expressed (DE) from the gene expression data (Fig 3, *Step 1*). This forms the ‘kernel set’. These TGs are then clustered based on their expression profiles (Fig 3, *Step 2*; algorithm outlined in Fig 4)–although they are DE in this context, they may differ in the directionality of regulation or be regulated by other TFs, thus putative TGs may be more closely correlated with a sub-cluster rather than the ‘average’ profile of the kernel set. Next, the other DE genes are compared to these clusters to derive ‘kernel set concordance’ (KSC) scores, which are used to predict new TGs for the TF (Fig 3, *Step 3*).

**Fig 5:**
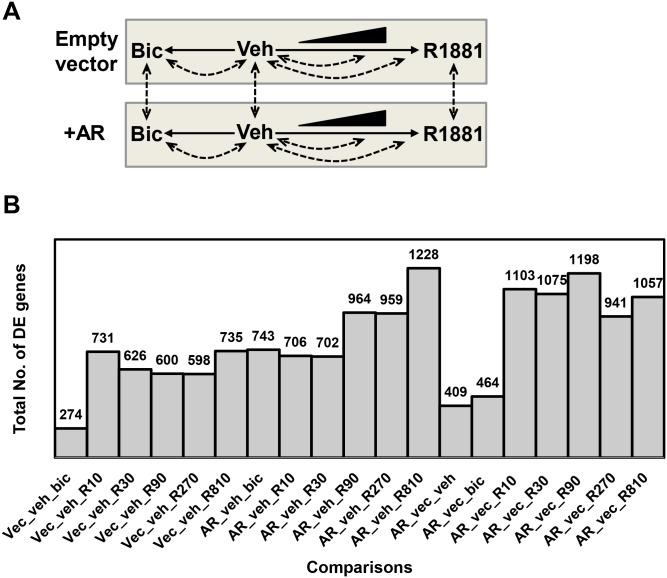
The androgen receptor (AR) as a case-study for Application II. **A)** Schematic view of the microarray experimental design in AR case study [14] and possible comparisons which can be used to obtain DE genes. **B)** The number of DE genes achieved by each comparison.

**Fig 6:**
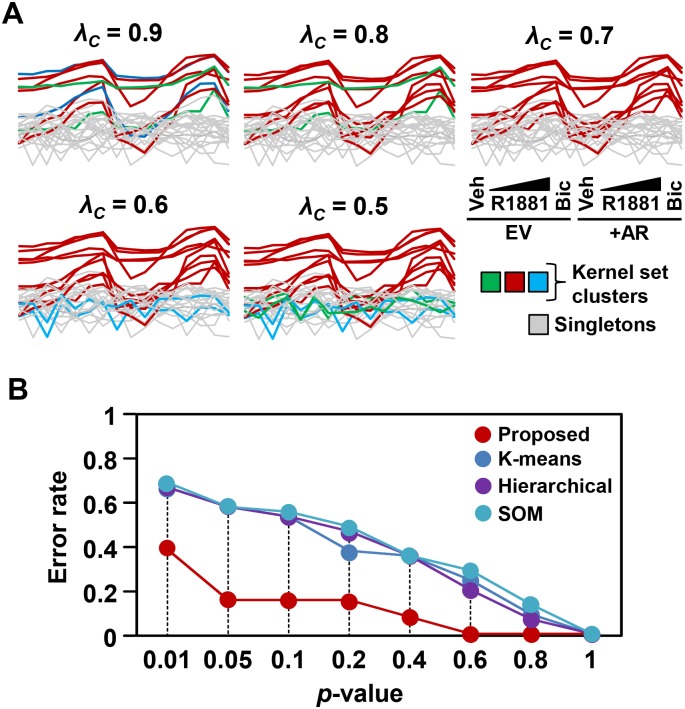
The kernel set clustering of androgen receptor (AR) target genes. **A)** Clusters identified within AR kernel set at different correlation thresholds. Different clusters are identified by different colours. Singletons are greyed out. **B)** Error rates of the recovery of AR known TGs (kernel set) at different *p*-value cut-offs using leave-one-out cross validation test. The proposed clustering algorithm was also replaced with commonly used clustering methods (i.e., K-means, hierarchical and self-organizing map) to illustrate the importance of the clustering component on the recovery of AR target genes.

We tested our approach of predicting novel TF-TG interactions using gene expression data in which the androgen receptor (AR) was manipulated both genetically (AR-overexpression) and pharmacologically (R1881 as an agonist, bicalutimide as an antagonist) (Fig 5A) [14]. We made 19 comparisons to obtain DE genes, namely the vehicle-treated conditions versus each drug treatment, and the empty-vector versus AR-overexpressing cells for the vehicle, bicalutamide, and maximum R1881 treatment (Fig 5A). Out of these comparisons, 4,114 DE genes were identified—from these, 30 DE genes were found as AR Rank 1 TGs in the ORTI database forming the kernel set (S1 File). The kernel set was then clustered using our proposed algorithm (Fig 4). Modulating the clustering threshold had a noticeable effect on the kernel set clusters, whereby lowering the threshold collapsed clusters with a similar behaviour into a single cluster and identified additional clusters (Fig 6A). Our clustering method requires the correlation threshold to be determined in advance. We selected a clustering threshold of *λ*_*c*_ = 0.6, with the correlation *p*-value ≤ 0.01. At this threshold, prevalent clusters were unambiguously distinguished from the outliers (singletons) (Fig 6A), which were removed prior to further analysis.

We initially verified the predictive power of this clustering method, and consequently the proposed concordance score, using leave-one-out cross validation (CV) over known AR TGs (i.e., kernel set genes). Each kernel gene was excluded from the kernel set, the remaining kernel genes clustered, and the KSC and *p*-value of the excluded kernel gene was computed. Kernel genes with KSC *p*-values less than a predetermined significance level were considered to be correctly classified as AR targets (true positives). In contrast, those with non-significant *p*-values were considered false-negatives, indicating the error rate. The error rate was 0.38 at *p*-value = 0.01 significance level, and decreased to only 0.15 at *p*-value = 0.05 (Fig 6B). To illustrate the advantage of using the proposed clustering algorithms as compared to commonly used clustering algorithms, we replaced the proposed clustering components with K-means, hierarchical and self-organizing map (SOM) clustering algorithms and compared the corresponding error rates using a similar CV analysis. In K-means and hierarchical clustering algorithms, we used the Calinski-Harabasz index [20] to identify the optimal number of clusters. SOM also requires the map dimensions to be defined by the user *a priori* [21]; we tried different dimensions and observed an insignificant variation in CV performance. Overall, the proposed clustering method outperformed these clustering algorithms in recovering the kernel genes at lower *p*-value cut-offs (Fig 6B).

The predictive performance of the KSC scores was also assessed using the receiver operating characteristic (ROC) curve analysis which plots the true positive rate (i.e., sensitivity) against the false-positive rate (i.e., 1-specificity) for different cut-off values of the KSC p-values. Here, the positive class comprised the AR kernel genes after removing the outliers (i.e., singletons), and the negative class included an equally-sized set of genes randomly selected out of the corresponding DE genes. The negative random set was resampled 100 times and the area under the curve (AUC) was computed. An AUC of 0.5 is produced by random selection, and thus any interesting classifier should have an AUC more than 0.5. We achieved an averaged AUC with 95% confidence interval of 0.9414∓0.0055 which demonstrates the predictive power of the proposed scoring mechanism.

Before searching for potential AR target genes, we narrowed down the list of candidate genes by focusing only on comparisons yielding the greatest biological effect sizes: Vehicle treatment versus largest R1881 dose, for both empty-vector and AR-overexpressed cells. These two comparisons yielded amongst the highest number of DE genes (Fig 5B), whilst reducing the diversity of comparisons and consequently the false positive rate. This generated 1,512 DE genes as candidates. Using our clustering algorithm and TG prediction method (Fig 3), we identified 146 genes whose expression profile bore a statistically significant correlation (KSC *p*-value < 0.05) to kernel set prevalent clusters (S1 File). An advantage of using gene expression data instead of motif-searching is that this immediately provides functional validation that a TF influences a putative TG’s expression in this particular biological context. However, there is a caveat, in that the predicted TGs may respond to AR modulation due to secondary effects. For instance, *LDLR* appears as a predicted TG, when the AR modulates *LDLR* expression by upregulating *SCAP* (a kernel set gene), the activator of *LDLR*’s TF, *SREBF2* [22, 23]. We consequently sought to overcome this limitation by employing the ranking system in ORTI: whilst Rank 1 TGs were used to provide the kernel set, we can provide preliminary validation of predicted TGs using the Rank 2 information, which consists primarily of HTP ChIP data. Within our 146 predicted AR TGs, 43.85% were found in the Rank 2 data.

**Fig 7:**
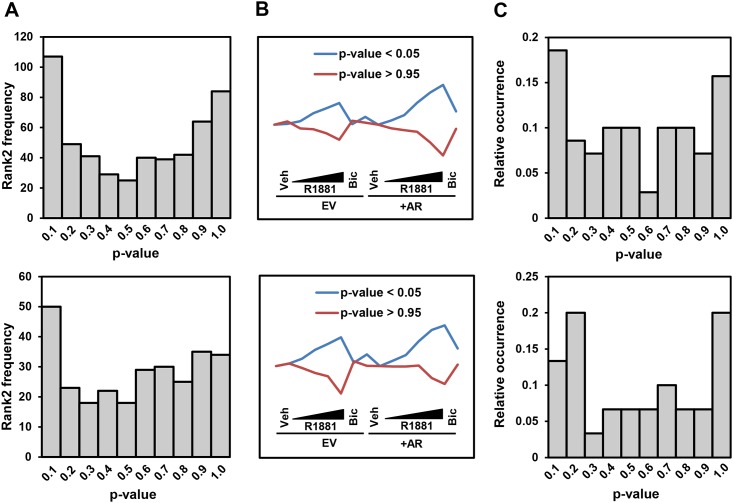
Rank 2 and functional validation of predicted androgen receptor (AR) TGs. The candidate differentially-expressed genes were sourced from two comparator groups: Vehicle treatment versus largest R1881 dose, for both AR-overexpressed (*top panels*) and empty-vector (*bottom panels*) cells. **A)** Binomial distributions of the spread of Rank 2 DE genes at different KSC *p*-values indicating that Rank 2 genes are more likely to appear at lower and higher ends of *p*-value spectrum. **B)** The average expression profiles of TGs at lower and higher ends of the *p*-value range (*p*-value < 0.05 or > 0.95). These two groups show a highly negative correlation—i.e., corr = -0.94 and -0.75 for top and bottom diagrams, respectively. **C)** Functional analysis of predicted TGs where density of occurrence of DE genes significantly representing AR function (as given in Table C of S3 File) is plotted at different KSC *p*-values; nearly similar binomial patterns as of Rank 2 analysis.

We hypothesised that amongst the DE genes, those that are Rank 2 TGs of AR are more likely to have lower KSC *p*-values (better concordance with the kernel set). However, instead of a power-law like distribution, we observed a binomial distribution, with the peaks at the lower and the higher ends of the *p*-value spectrum (Fig 7A). The accumulation of Rank 2 TGs at high-*p*-values was unexpected. Indeed, the expression pattern of this group of TGs is extremely negatively correlated to those with low KSC *p*-values (Fig 7B). This raises the hypothesis that in this context, the AR regulates a novel set of genes in an opposite manner to the Rank 1 TGs (and prevalent kernel set clusters). For instance, the prostate consists of a minor population of neuroendocrine cells. It is well-established that AR represses differentiation to the neuroendocrine phenotype, with androgen deprivation leading to the adoption of neuronal markers [24]. This transdifferentiation has been shown to be driven by protein tyrosine phosphatases, which play a key role in neuronal development [25], including PTPRA [26] and PTPB1 [27]. Here, we found PTPRB (KSC *p*-value 0.937) and PTPRR (KSC *p*-value 0.939)—both are involved in neuronal differentiation [28, 29] and thus may play a role in neuroendocrine differentiation. In addition, it is known that E-cadherin is downregulated by AR [30], and here we find its binding partner, CDH3 [31], may also be repressed directly by AR (KSC *p*-value = 0.957). Another interesting target is dopa decarboxylase (KSC *p*-value = 0.893), a risk factor for recurrence following androgen ablation therapy [32]. Thus, considering high KSC *p*-value genes, particularly when enriched amongst the Rank 2 data, may also reveal novel genes.

We performed a similar analysis looking at functionality, examining whether the functions of the DE genes with lower *p*-values are enriched for those of the AR Rank1 TGs (i.e., kernel set). Using MSigDB [33] pathways (KEGG, Biocarta, and Reactome) and Gene Ontology sets (molecular functions and biological processes), we identified 15 functional terms significantly enriched (FDR *q*-value < 0.05) by AR kernel genes (Table C in S3 File). Then, for each DE gene, we calculated a hypergeometric *p*-value indicating whether the functions of AR kernel genes are overrepresented by the gene’s functions. We then plotted the histogram of the relative occurrences of AR target functions within the list of DE genes ranked by the KSC *p*-value (kernel genes were excluded to avoid circular argument). We observed a similar pattern as obtained using Rank 2 analysis: binomial distributions (Fig 7C) indicating that AR functions are more likely to be enriched by DE genes at the lower and higher ends of the KSC *p*-value spectrum. This supports our hypothesis that AR regulates a novel set of genes in an opposite manner to the Rank 1 TGs (i.e. down-regulated by R1881 and up-regulated by bicalutamide, Fig 7B).

**Fig 8:**
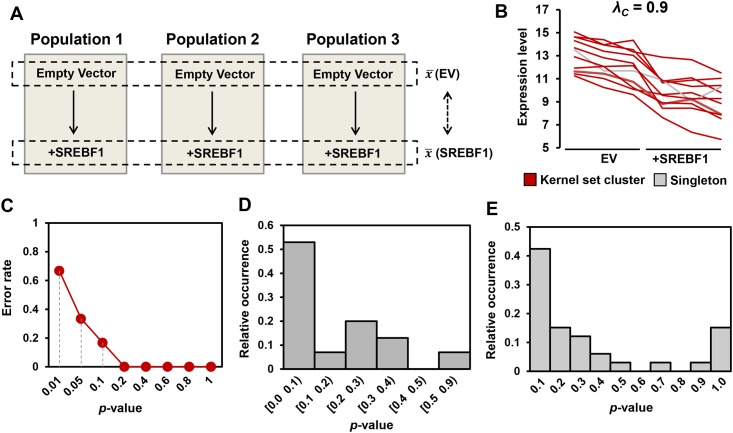
The sterol-regulatory element binding factor (SREBF1) as a case-study for Application II. **A)** Microarray experimental setup in SREBF1 case study. **B)** One singleton and one prevalent cluster were identified within SREBF1 kernel set using a clustering threshold of *λ*_*c*_ = 0.9 corresponding to the correlation *p*-value ≤ 0.01. **C)** Error rates of the recovery of SREBF1 kernel genes at different *p*-value cut-offs using leave-one-out cross validation test. **D)** Rank 2 analysis: a power-law like distribution indicates that a majority of putative SREBF1 TGs correlate with the prevalent kernel set clusters. **E)** Functional analysis: power-law like distribution suggests that DE genes that possessed the enriched functions of the kernel set genes (Table D in S3 File) were likely to have lower KSC *p*-values.

This study contained genetic, pharmacological and dose-curve elements, providing many points of comparison for our clustering analysis. To determine if such an intricate, multi-dimensional experimental design is required, we applied our pipeline to a simpler dataset. We analysed a microarray study in which sterol regulatory element binding transcription factor 1 (SREBF1) was over-expressed in three separate populations of muscle cells [16] (Fig 8A). From 256 DE genes, 11 TGs of SREBF1 were included in the kernel set (S1 File). Using a clustering threshold of *λ*_*c*_ = 0.9 corresponding to the correlation *p*-value ≤ 0.01, we identified one prevalent cluster where 1 TG is excluded as a singleton (Fig 8B). Subsequently, 51 other DE genes showed a statistically significant correlation with these kernel set genes (KSC *p*-value < 0.05) (S1 File). To evaluate the predictive power of the concordance scores, we applied leave-one-out cross validation on SREBF1 kernel genes: 66.67% of kernel genes were correctly recovered at *p*-value = 0.05. The recovery rate sharply improved to 100% when the significance cut-off shifts to 0.2 (Fig 8C). Additionally, we estimated the sensitivity and specificity of the prediction at different KSC *p*-value cut-offs using the SREBF1 kernel set as positives and an equally-sized set of random DE genes as negatives. We resampled the negative set 100 times and computed the area under the ROC curve; an average AUC (with 95% confidence interval) of 0.8248∓0.0160 was achieved which confirms the performance of the proposed KSC-based prediction.

Applying the Rank 2 data, we found that the spread of KSC *p*-values of the DE Rank 2 TGs appeared similar to a power-law distribution (Fig 8D), suggesting that a majority of putative SREBF1 TGs correlate with the prevalent kernel set clusters. Furthermore, DE genes that possessed the enriched functions of the kernel set genes (Table D in S3 File) were likely to have lower KSC *p*-values (Fig 8E). Overall, this supports the notion that a simpler experimental design (with a single dimension) can also be used to predict TGs.

## Conclusions

In this study, we constructed a new online, publicly-available TF-TG database, ORTI. Combining existing databases and additional studies from the literature, data has been incorporated from a range of mammalian species (primarily human, rat, and mouse), and cell-types, in an attempt to overcome bias from any individual context (e.g. inflammation from NFI-Regulome, [9]). A significant portion of TFs are represented in ORTI, but additional TFs can be incorporated in the future through online user submissions and additional literature searches. While ORTI alone serves as a repository for experimentally-validated TF-TG interactions, we developed two tools for interrogating gene expression data as examples of ORTI applications: 1) the identification of TFs modulated in response to a stimulus, and 2) the prediction of novel TF-TG interactions. These tools are available to download (http://orti.sydney.edu.au/download.html) which allows users to customise their own settings, to adopt the algorithms for other contexts (e.g., kinase-substrate interactions in phosphoproteomics data), or to incorporate the algorithms into their own workflows. In addition, ORTI can be used in other applications, such as quantitative analysis of TF dynamics [34], genomic analysis of regulatory network dynamics [35] or analysis of network motifs in transcriptional regulatory networks [36]. ORTI can also serve as a rich ‘gold standard’ for computational modelling of regulatory networks [37].

**For Application 1**, we used ORTI to identify TFs regulated in the expression data, using the commonly used hypergeometric enrichment analysis [4, 33]. ORTI outperformed the existing databases, regardless of using either all of the TF-TG data (Table 2) or just the Rank 1 data (Table A of S3 File). This can be attributed to its greater coverage of TFs and TF-TG interactions (Table 1). An interesting potential improvement could be the incorporation of sign-sensitivity. For instance, the TFactS database records whether a TF upregulates or downregulates a TG, although this has been shown not to be required for identifying TFs [4]. Our results also indicate that the success of this application depends on the information available about the TF within ORTI, with the possibility of more obscure TFs not being detected in GE data (e.g., PDX1, Table 2). Thus, we intend to periodically update ORTI with novel, experimentally-validated TF-TG interactions from the literature.

Furthermore, we demonstrated that these pairwise TF-TG interactions can reveal the topology of the transcriptional network. We applied this to adipogenesis: by utilising a dataset that measured gene expression changes over time, we showed that this biological process consists of a cascade of transcriptional interactions (Fig 2). This is highly nonlinear in nature, highlighting the need to distinguish between primary and secondary effects when constructing such networks.

**For Application 2**, we aimed to identify novel TF-TG interactions, key to constructing cellular regulatory processes and understanding how these become dysregulated in disease settings. Indeed, TF-TG interactions are in some cases context-dependent, in that the TF may regulate a specific TG in one experimental context but not others. This is influenced by a range of cell-specific factors such as the expression of coregulators, ligands and receptors that may influence particular TFs in unique ways, or the co-regulation of alternate TFs that may cross talk with other TFs in the network. Unique to our database, we can predict context-specific interactions on different experimental complexities. Our approach involved comparing the expression patterns of DE genes to that of the kernel set, known targets of the TF that are DE in the context of interest. This is similar to the method developed by Mrowka *et al*. [38] to discover novel TGs of NF-kβ. However, their kernel set was only a single cluster overlooking the possibility of heterogeneity in TG expression profiles. Furthermore, they followed a context-generic approach by sourcing over 1,200 microarray experiments with the aim of exploring the vast amount of expression information available in public databases, whilst our goal is to uncover novel interactions within a specific experiment/context.

Amongst the diverse statistical or computational models for predicting TF-TG interactions, there is a growing trend to combine other sources of information with the gene expression data to enhance the prediction accuracy—e.g., gene expression data and prior knowledge for context-specific TG prediction in a Bayesian statistical model [39], motifs with expression data using a binary classification model [40] or conserved motifs’ patterns and positions as features for support vector machine (SVM) classifier [41], and gene expression with ChIP data to predict Pou5f1 targets [42]. Xu *et al*. [43] generated a TF-TG similarity matrix by integrating gene expression data with gene ontology similarity analysis, promoter motif searching, protein interaction and literature mining.

Here, we used ORTI Rank 1 and 2 data to predict and validate context-TF-TG interactions. This includes two complementary approaches: (1) comparing DE genes to the kernel set and using Rank 2 data as a filter, to obtain a high-quality set of predicted genes that behave like the kernel set, and (2) comparing DE to Rank 2 data, then to the kernel set, which may yield novel TGs that are regulated differently from the kernel set (Fig 6A). Indeed, the novel subset of AR genes will be investigated in future experiments. In both prediction approaches, the predicted TGs may be paired with a motif searching program (e.g., JASPAR [2], which is publicly available) to provide another level of validation filter.

Overall, we have constructed a new TF-TG database, and applied it to gene expression data to identify and predict transcriptional interactions, demonstrating its potential value in unravelling the topology of transcriptional networks. Given that many TFs are ligand-inducible, understanding this biology can inform therapeutic options for related diseases. For instance, identifying novel TGs of the AR can provide further insight into how the AR drives prostate physiology and prostate cancers become resistant to androgen ablation therapy. These applications, along with a query form to the database, are incorporated into an online user interface (http://orti.sydney.edu.au).

## Materials and Methods

### Database construction

To build the ORTI database, we retrieved mammalian TFs and their associated TGs from publicly available databases of TF-TG interactions, namely HTRI [10], TFactS [4, 44], TRED [6, 8], TRRD [45], PAZAR [7, 46], and NFI-Regulome [9], as well as from the literature using PubMed searches for TFs of interest (Fig 1A). Gene names were disambiguated by consulting with NCBI to include the official symbol of the gene for the specified species as provided by the HGNC (HUGO Gene Nomenclature Committee). We clarified any ambiguous cases by manual curation, consulting the original articles to determine the relevant TFs and TGs under examination, after which any remaining ambiguous cases were discarded. For transcriptional heterodimers (e.g. AP1), the TG was assigned to the individual TF components where possible (e.g. Fos, Jun). However, when the information source did not specify these TFs, the original heterodimer name was retained. Gene symbol synonyms and gene IDs were compiled from NCBI by matching the symbol and species pairs.

For each TF-TG interaction, ORTI provides: 1) the symbols of the TF and the TG; 2) the synonyms of TF and TG symbols; 3) TF and TG Entrez IDs; 4) TF and TG accession identifiers in multiple reference databases; 5) the corresponding species; 6) the reference information, including the database containing the interaction information; 7) the accession ID/PubMed ID; 8) the experimental technique used to detect the TF-TG interaction; and 9) the reliability-rank of this technique. We incorporated this rank since many existing databases do not clearly separate out TF-TG interactions validated by LTP experimental methods from those derived by HTP screening techniques. This feature categorizes TF-TG interactions into three ranks: ‘1’ for interactions validated by LTP techniques, ‘2’ for those characterised from HTP screenings, and ‘3’ for those predicted by promoter-sequence conservation or differential expression of genes in response to manipulating a TF (without further LTP validation to confirm this is not a secondary effect). If the supporting evidence for a TF-TG interaction was not provided by a database or was ambiguous (for instance, chromatin immunoprecipitation can be used as both as HTP and LTP technique), manual curation of the literature was performed to clarify the ambiguity. The experimental evidence sufficient for each level of our ranking system is detailed in S2 File. The composition of the ORTI database is depicted in Fig 1. We find that overall, no single database dominates ORTI.

The online version of ORTI database is implemented in JavaScript, a high-level programming language supported by most web browsers and widely used to add interactive features and dynamic content to the web sites. The database arrays and interface functions are embedded together in a single web page that processes search requests locally on the client computer. This approach is more sensitive to the client's hardware and software configuration than the more common client-server model, but it offers many advantages such as immediate access to the entire database required for real-time interface features, autonomous offline operation, simplicity of the code, stable performance and secure server configuration.

The database is represented in JavaScript as a two-dimensional matrix where textual values are replaced with their indices in the list of unique values associated with each parameter. The database search routine is executed every time the user changes the content of the input fields or alters state of the filters. If the previous search is not finished the new call cancels it. In the first phase of the search, the code marks unique TF and TG names/aliases that match search keywords. In the second phase all records in the database are enumerated and those matching the search criteria are gathered together. Lastly, the records are presented to the user as a formatted table. This algorithm is fast enough to enable the incremental search feature without any optimizations which are usually timely inefficient.

Additionally, the interface provides batch search of multiple keywords, suggests for partially matching names, identifies missing names, and allows user to filter records by species, contributor and rank. The interface also offers options in searching by either gene ID or gene name for both TFs and TGs. The user can select search results by clicking a button, copy data to the clipboard and paste into any spreadsheet software. The current implementation of the interface is best suited for finding the intersection of a set of TFs with a set of TGs. Large lookups of either TF or TG names with thousands of matching records can slow down browser's rendering pipeline and might be more convenient to perform within the master database which can be downloaded in Excel format from the web site (http://orti.sydney.edu.au/download.html).

### Application I: Prediction of context-specific transcription factors

To identify TFs modulated in a particular context, we performed a TF enrichment analysis on a list of differentially expressed (DE) genes within that context. Accordingly, for each TF, the number of TGs in ORTI and input gene list were compared using the right-sided Fisher’s exact test where the *p*-value for the null hypothesis is computed based the hypergeometric distribution:

$$p=\frac{1}{\left(\begin{array}{c} N\\ n \end{array}\right)}\overset{i=\;n}{\underset{i=k}{\sum }}\left(\begin{array}{c} n\\ i \end{array}\right)\left(\begin{array}{c} N-K\\ n-i \end{array}\right),$$

where *N* is the total number of TF-TG interactions in ORTI, *n* is the number of input genes, *K* is the total number of TGs annotated as being regulated by a TF in ORTI, and *k* is the number of input genes annotated as the TF’s targets in ORTI. Since multiple TFs are tested, the nominal *p-*value was adjusted for multiple hypothesis tests using Bonferroni correction.

### Application II: Prediction of novel context-specific TF target genes

To predict novel TF-TG relationships, we examined differentially expressed genes, assessing how well their expression patterns correlated with known TGs of our TFs of interest. The pipeline for this analysis (Fig 3) requires the user’s gene expression data—i.e., the expression profiles of DE genes—and TFs of interest, the latter identified either by the user or Application I. The pipeline involves three steps:

#### 1. Acquire the kernel set

The set of Rank 1 TGs of the queried TF is first retrieved using ORTI. We define a *kernel set* to be the set of the TF targets which are significantly deregulated under the given condition. So, if *G*_*de*_ = {*g*_1_, ⋯, *g*_*m*_} is the set of differentially expressed genes, and *TG*_*tf*_ = {*tg*_1_, ⋯, *tg*_*n*_} is the set of the TF target genes retrieved out of ORTI, then the kernel set is the intersection of these two sets, i.e., *G*_*k*_ = *TG*_*tf*_ ∩ *G*_*de*_.

#### 2. Cluster genes within the kernel-set

For the remaining genes, *g*_*i*_ ∈ *G*_*de*_ − *G*_*k*_, we aim to calculate their concordance with the set of kernel genes *G*_*k*_. However, *G*_*k*_ may be heterogeneous in expression patterns—factors such as directionality, regulation by other TFs, and time-dependence can generate diversity of gene expression patterns within the kernel set. Thus, genes that correlate poorly with the entire kernel set may at most be highly correlated with only a subset of *G*_*k*_. Accordingly, these subsets of co-regulated kernel genes are identified using a clustering algorithm.

Commonly-used clustering algorithms (e.g., K-means, hierarchical, and self-organizing map (SOM)) hold some assumptions or constraints which make them unsuitable for this application. For instance, they often assume a user-defined number of clusters (or map grids in case of SOM) while the number of co-regulated kernel genes is not *a priori* known and should be determined on the fly. These algorithms also classify all data-points including outliers into at least one cluster which may adversely affect the clusters’ dominant patterns. We, however, are interested in distinguishing the prevalent expression patterns of kernel genes from those of the outliers. Furthermore, the clustering algorithms are usually designed to group data points into two or more clusters, overlooking the situation when all the kernel genes follow relatively similar expression pattern forming a single cluster.

Consequently, we developed a customised clustering algorithm (Fig 4). This algorithm groups genes whose reciprocal correlation value is above a given stringent cut-off threshold, iteratively relaxes the cut-off threshold, and merges the clusters accordingly until reaching a *critical* correlation cut-off threshold. The initial correlation cut-off *λ*_0_ ≤ 1.0, should be large enough to avoid missing clusters of highly correlated genes. In our experiments, we set it to be 0.9 as it is an upper-bound of kernel genes’ mutual absolute correlation values. On the other hand, the reduction step size *ε* > 0 should be small enough to ensure the placement of each kernel gene into the best co-regulated cluster. We chose it to be 0.05. Smaller values for step size or larger values for the initial correlation do not significantly affect the prediction performance, although it may slightly de-accelerate the algorithmic rate. The key parameter, however, is the critical correlation *λ*_*c*_ < 1 which can either produce unnecessarily-high singletons or place heterogeneous genes in the same cluster if it is chosen to be very large or small, respectively. We observed that the performance should be reasonably stable if *λ*_*c*_ is set to a value such that the corresponding correlation *p*-value ≤ 0.01. Once the clustering is completed, singleton clusters (i.e., outliers) can either be retained or removed from the subsequent analysis. In our experiments, we considered outliers as noise in kernel set expression values, and removed them prior to further analysis.

#### 3. Calculate the concordance of DE genes

Using the proposed clustering algorithm, the kernel genes are grouped into *K* clusters *C*_1_, *C*_2_…, *C*_*K*_, where *c*_*l*_ denotes the centroid of cluster *C*_*l*_. We then define the *cluster-based concordance* of a DE gene *g*_*i*_ with cluster *C*_*l*_ as the Pearson correlation of the expression vectors of *g*_*i*_ and the cluster’s centroid *c*_*l*_—i.e., *δ*_*l*_(*g*_*i*_, *C*_*l*_) = *corr*(*g*_*i*_, *c*_*l*_). The *kernel-set concordance* (KSC) is then defined to be the maximum value of the cluster-based concordance measures—i.e., *δ*(*g*_*i*_, *G*_*k*_) = max(*δ*_1_(*g*_*i*_, *C*_1_), ⋯, *δ*_*l*_(*g*_*i*_, *C*_*l*_)) —implying that a gene is considered to be in concordance with the kernel set if it shows high correlation with at least one cluster of co-regulated target genes. The schematic view of the calculation of *kernel-set concordance* is illustrated by Fig 3.

In order to assess the significance of the kernel-set concordance scores, for each KSC, a nominal *p*-value of the null hypothesis is estimated using the distribution of KSC scores under the null hypothesis that gene labels does not matter. The null distribution is derived by 10,000 iterations of a permutation test procedure: randomly permuting the gene labels across the entire microarray dataset, re-computing the KSC scores, and then drawing a random gene’s score. The one-sided *p*-value of the observed KSC score is then calculated as the proportion of sampled permutations where the KSC score was greater than or equal to the observed score. The set of differentially expressed genes are sorted by *p*-value, with top-ranked genes passing a chosen significance level being considered putative targets of the input TF.

## Supporting Information

S1 File List of kernel set TGs and DE genes with KSC *p*-value < 0.05 (i.e., potential novel TGs) in AR and SREBF1 case studies. (XLSX)

S2 File Rankings of the biochemistry techniques used to detect TF-TG interactions included in ORTI. (PDF)

S3 File Performance of ORTI as compared to other TF-TG interaction databases in identifying modulated TFs when just the Rank 1 data were considered (Table A); the parameters of Table 2 enrichment tests (Table B); functional terms enriched by TGs included in kernel sets of AR (Table C) and SREBF1 (Table D) using MSigDB; details of preprocessing and differential expression analyses for the adipogenesis time-course data used as a test case for Application 1 (Fig A). (PDF)

## Abbreviations

AR: Androgen receptor
AUC: Area under the curve
ChIP: Chromatin immunoprecipitation
CV: Cross validation
DE: Differentially expressed
FDR: False discovery rate
GE: Gene expression
HTP: High-throughput
KSC: Kernel set concordance
LTP: Low-throughput
ORTI: Open-access repository of transcriptional interactions
ROC: Receiver operating characteristic
SOM: Self organising map
SREBF1: Sterol regulatory element binding transcription factor 1
TF: Transcription factor
TG: Target gene
