# Supplementary material for: ORTI: An Open-Access Repository of Transcriptional Interactions for Interrogating Mammalian Gene Expression Data
Source: PLoS One. 2016 Oct 10;11(10):e0164535. doi: 10.1371/journal.pone.0164535 (PMC5056720; doi:10.1371/journal.pone.0164535)
Supplement: S2 File — (PDF) [file pone.0164535.s002.pdf]

Rankings of the techniques used to detect TF-TG interactions included in ORTI.

Rank	Biochemistry Technique
1	AVIDIN/BIOTIN-CONJUGATED DNA BINDING ASSAY
1	CONCATENATE CHROMATIN IMMUNOPRECIPITATION
1	DNA AFFINITY CHROMATOGRAPHY
1	DNA AFFINITY PRECIPITATION ASSAY
1	ELECTROPHORETIC MOBILITY SHIFT ASSAY
1	ELECTROPHORETIC MOBILITY SHIFT ASSAY
1	EXPERT CURATED::LITERATURE DERIVED
1	MUTAGENESIS
1	PROTEIN BINDING ASSAY::DNA-PROTEIN PRECIPITATION ASSAY
1	PROTEIN BINDING ASSAY::DNASE FOOTPRINTING ASSAY
1	PROTEIN BINDING ASSAY::METHYLATION INTERFERENCE ASSAY
1	PROTEIN BINDING ASSAY::UNKNOWN
1	REPORTER GENE ASSAY::CHLORAMPHENICOL ACETYLTRANSFERASE (CAT) ASSAY
1	REPORTER GENE ASSAY::LUCIFERASE ASSAY
1	REPORTER GENE ASSAY::GFP EXPRESSION ASSAY
1	REPORTER GENE ASSAY::LACZ EXPRESSION ASSAY
1	REPORTER GENE ASSAY::RABBIT BETA-GLOBIN EXPRESSION ASSAY
1	PROTEIN BINDING ASSAY:: (SOUTHWESTERN BLOTTING)
1	SURFACE PLASMON RESONANCE
2	CHROMATIN IMMUNOPRECIPITATION::COUPLED WITH DEEP SEQUENCING
2	CHROMATIN IMMUNOPRECIPITATION::COUPLED WITH MICROARRAY
2	CHROMATIN IMMUNOPRECIPITATION::CPG
2 or 1*	CHROMATIN IMMUNOPRECIPITATION
2	CHROMATIN IMMUNOPRECIPITATION::COUPLED WITH TAG SEQUENCING
2	CHROMATIN IMMUNOPRECIPITATION::STREPTAVIDIN
2	REPORTER GENE ASSAY::YEAST ONE-HYBRID ASSAY
3	RNA EXPRESSION ASSAY::RNASE PROTECTION ASSAY (RPA)
3	RNA EXPRESSION ASSAY::MICROARRAY
3	SEQUENCE CONSERVATION::CO-EXPRESSED GENE CONSERVATION
3	SEQUENCE CONSERVATION::CONSERVATION FOUND BY ALIGNMENT
3	SEQUENCE CONSERVATION::ORTHOLOGOUS GENE CONSERVATION

* The source database reported these studies as “ChIP” without any further details, any study with >5 hits was expertly curated.
