# Supplementary material for: ORTI: An Open-Access Repository of Transcriptional Interactions for Interrogating Mammalian Gene Expression Data
Source: PLoS One. 2016 Oct 10;11(10):e0164535. doi: 10.1371/journal.pone.0164535 (PMC5056720; doi:10.1371/journal.pone.0164535)
Supplement: S3 File — (PDF) [file pone.0164535.s003.pdf]

Table A: ORTI outperforms other databases in identifying modulated TFs in gene expression data. This analysis was performed identically to that described in Table 2, except using only Rank 1 data; p-values and and Bonferroni corrected p-values are shown. '—' means that the TF was not found in the database.

		Single TF modulated				Biological process			
**Database**		**AR**	**SREBF1**	**E2F1**	**PDX1**	**CEBPA**	**CEBPB**	**CEBPD**	**PPARG**
**ORTI**	*p*-value	3.44E-10	9.48E-20	1.56E-16	8.77E-06	1.56E-08	1.04E-07	7.56E-06	2.45E-07
	Adj *p*-value	1.35E-07	3.72E-17	6.12E-14	3.44E-03	6.11E-06	4.06E-05	2.96E-03	9.59E-05
**HTRI**	*p*-value	0.111	1	0.0270	2.31E-04	0.175	0.0613	0.0691	0.438
	Adj *p*-value	1	1	1	0.0340	1	1	1	1
**NFI-Reg**	*p*-value	—	—	—	—	—	—	0.0458	—
	Adj *p*-value	—	—	—	—	—	—	0.824	—
**PAZAR**	*p*-value	1	—	—	1.25E-05	1	0.293	1	—
	Adj *p*-value	1	—	—	1.76E-03	1	1	1	—
**TFactS**	*p*-value	6.22E-07	1.25E-12	3.60E-05	1.77E-02	4.88E-06	7.59E-04	0.0263	2.54E-05
	Adj *p*-value	1.47E-04	2.96E-10	8.51E-03	1	1.15E-03	0.179	1	6.01E-03
**TRED**	*p*-value	0.0108	—	6.86E-03	—	3.86E-06	1.05E-03	1.20E-03	1.71E-04
	Adj *p*-value	1	—	0.816	—	4.59E-04	0.125	0.143	0.0204
**TRRD**	*p*-value	0.0543	5.63E-04	1	1	0.0426	0.0426	0.126	0.491
	Adj *p*-value	1	0.0945	1	1	1	1	1	1

Table B: The parameters of the hypergeometric tests conducted in **Table 2** of the main text. **M** is the total number of TF-TG interactions in a database. **N** is number of differentially expressed genes in the database. **K** is the total number of TGs annotated as being regulated by the TF in the database, and **x** is the number of differentially expressed genes annotated as the TFs' targets in the database; '—' implies that the TF was not found in the database.

DB \ TF		Single TF modulated				Biological process			
		**AR**	**SREBF1**	**E2F1**	**PDX1**	**CEBPA**	**CEBPB**	**CEBPD**	**PPARG**
**ORTI** M = 72817	N	3605	219	808	51	1618	1618	1618	1618
	K	6676	1221	990	50	378	166	53	132
	x	1468	32	46	2	46	28	13	23
**HTRI** M = 51872	N	3466	205	744	43	1568	1568	1568	1568
	K	6596	4	44	2	16	24	5	6
	x	1443	0	4	1	3	5	2	1
**NFI-Reg** M = 200	N							11	
	K	—	—	—	—	—	—	1	—
	x							1	
**PAZAR** M = 8449	N	1134		257	22	534	534	534	
	K	1	—	124	39	109	5	1	—
	x	0		8	2	7	1	0	
**TFactS** M = 6727	N	972	65	168	12	358	358	358	358
	K	58	51	161	10	117	71	23	41
	x	24	11	13	1	19	11	4	10
**TRED** M = 9323	N	991		190		392	392	392	392
	K	80	—	701	—	237	116	42	119
	x	33		25		35	20	10	20
**TRRD** M = 1305	N	250	16	46	9	82	82	82	82
	K	8	14	8	8	21	21	2	10
	x	4	3	0	0	4	4	1	1

Table C: Functional terms enriched by AR kernel targets using MSigDB; hypergeometric test is used for assessing the statistical significance.

Gene Set Name	Description	FDR q-value
REACTOME ANDROGEN BIOSYNTHESIS	Genes involved in Androgen biosynthesis	7.63E-05
REACTOME STEROID HORMONES	Genes involved in Steroid hormones	1.15E-03
REACTOME METABOLISM OF STEROID HORMONES AND VITAMINS A AND D	Genes involved in Metabolism of steroid hormones and vitamins A and D	1.37E-03
REACTOME HORMONE LIGAND BINDING RECEPTORS	Genes involved in Hormone ligand-binding receptors	9.96E-03
MALE GONAD DEVELOPMENT	The process whose specific outcome is the progression of the male gonad over time, from its formation to the mature structure.	9.96E-03
REACTOME GLYCOPROTEIN HORMONES	Genes involved in Glycoprotein hormones	9.96E-03
KEGG PROSTATE CANCER	Prostate cancer	9.96E-03
REACTOME PEPTIDE HORMONE BIOSYNTHESIS	Genes involved in Peptide hormone biosynthesis	1.18E-02
REACTOME REGULATION OF INSULIN LIKE GROWTH FACTOR IGF	Genes involved in Regulation of Insulin-like Growth Factor (IGF) Activity by Insulin-like Growth Factor Binding Proteins (IGFBPs)	1.39E-02
REACTOME G ALPHA S SIGNALLING EVENTS	Genes involved in G alpha signalling events	1.74E-02
DEVELOPMENT OF PRIMARY SEXUAL CHARACTERISTICS	The process of the progression of the primary sexual characteristics over time, from their formation to the mature structures. The primary sexual characteristics are the testes in males and the ovaries in females	2.85E-02
CELL CELL SIGNALING	Any process that mediates the transfer of information from one cell to another	2.85E-02
REPRODUCTIVE PROCESS	A biological process that directly contributes to the process of producing new individuals by one or two organisms.	3.18E-02
KEGG FRUCTOSE AND MANNOSE METABOLISM	Fructose and mannose metabolism	4.14E-02
REACTOME METABOLISM OF LIPIDS AND LIPOPROTEINS	Genes involved in Metabolism of lipids and lipoproteins	4.33E-02

Table D: Functional terms enriched by SREBF1 kernel targets using MSigDB; hypergeometric test is used for assessing the statistical significance.

Gene Set Name	Description	FDR q-value
OXIDOREDUCTASE ACTIVITY	Catalysis of an oxidation-reduction (redox) reaction, a reversible chemical reaction in which the oxidation state of an atom or atoms within a molecule is altered.	4.51E-04
LIPID METABOLIC PROCESS	The chemical reactions and pathways involving lipids, compounds soluble in an organic solvent but not, or sparingly, in an aqueous solvent. Includes fatty acids; neutral fats, other fatty-acid esters, and soaps; long-chain (fatty) alcohols, etc.	5.40E-04
LIPID BIOSYNTHETIC PROCESS	The chemical reactions and pathways resulting in the formation of lipids, compounds soluble in an organic solvent but not, or sparingly, in an aqueous solvent.	8.24E-04
CELLULAR CARBOHYDRATE METABOLIC PROCESS	The chemical reactions and pathways involving carbohydrates, any of a group of organic compounds based of the general formula Cx(H2O)y, as carried out by individual cells.	1.17E-03
TRICARBOXYLIC ACID CYCLE INTERMEDIATE METABOLIC PROCESS	The chemical reactions and pathways involving intermediates of the tricarboxylic acid cycle.	1.17E-03
CARBOHYDRATE METABOLIC PROCESS	The chemical reactions and pathways involving carbohydrates, any of a group of organic compounds based of the general formula Cx(H2O)y.	2.20E-03
CARBOXYLIC ACID METABOLIC PROCESS	The chemical reactions and pathways involving carboxylic acids, any organic acid containing one or more carboxyl (COOH) groups or anions (COO-).	2.20E-03
ORGANIC ACID METABOLIC PROCESS	The chemical reactions and pathways involving organic acids, any acidic compound containing carbon in covalent linkage.	2.20E-03
CELLULAR LIPID METABOLIC PROCESS	The chemical reactions and pathways involving lipids, as carried out by individual cells.	5.34E-03
COENZYME METABOLIC PROCESS	The chemical reactions and pathways involving coenzymes, any of various nonprotein organic cofactors that are required, in addition to an enzyme and a substrate, for an enzymatic reaction to proceed.	6.66E-03
PHOSPHOLIPID BIOSYNTHETIC PROCESS	The chemical reactions and pathways resulting in the formation of phospholipids, any lipid containing phosphoric acid as a mono- or diester.	6.66E-03
NUCLEOTIDE METABOLIC PROCESS	The chemical reactions and pathways involving a nucleotide, a nucleoside that is esterified with (ortho)phosphate or an oligophosphate at any hydroxyl group on the glycose moiety; may be mono-, di- or triphosphate; this definition includes cyclic-nucleotides (nucleoside cyclic phosphates).	7.28E-03
MEMBRANE LIPID BIOSYNTHETIC PROCESS	The chemical reactions and pathways resulting in the formation of membrane lipids, any lipid found in or associated with a biological membrane.	9.38E-03
NUCLEOBASENUCLEOSIDE AND NUCLEOTIDE METABOLIC PROCESS	The chemical reactions and pathways involving nucleobases, nucleosides and nucleotides.	1.00E-02
COFACTOR METABOLIC PROCESS	The chemical reactions and pathways involving a cofactor, a substance that is required for the activity of an enzyme or other protein. Cofactors may be inorganic or organic, in which case they are referred to as coenzymes.	1.03E-02
PHOSPHOLIPID METABOLIC PROCESS	The chemical reactions and pathways involving phospholipids, any lipid containing phosphoric acid as a mono- or diester.	1.76E-02
ALCOHOL METABOLIC PROCESS	The chemical reactions and pathways involving alcohols, any of a class of alkyl compounds containing a hydroxyl group.	2.38E-02
MEMBRANE LIPID METABOLIC PROCESS	The chemical reactions and pathways involving membrane lipids, any lipid found in or associated with a biological membrane.	3.00E-02
EACTOME METABOLISM OF LIPIDS AND LIPOPROTEINS	Genes involved in Metabolism of lipids and lipoproteins	5.3 e-9
REACTOMEFATTYACID TRIACYLGLYCEROL AND KETONE BODY METABOLISM	Genes involved in Fatty acid, triacylglycerol, and ketone body metabolism	5.11 e-3
KEGG BIOSYNTHESIS OF UNSATURATED FATTY ACIDS	Biosynthesis of unsaturated fatty acids	5.28 e-3
KEGG PPAR SIGNALING PATHWAY	PPAR signaling pathway	4 e-2

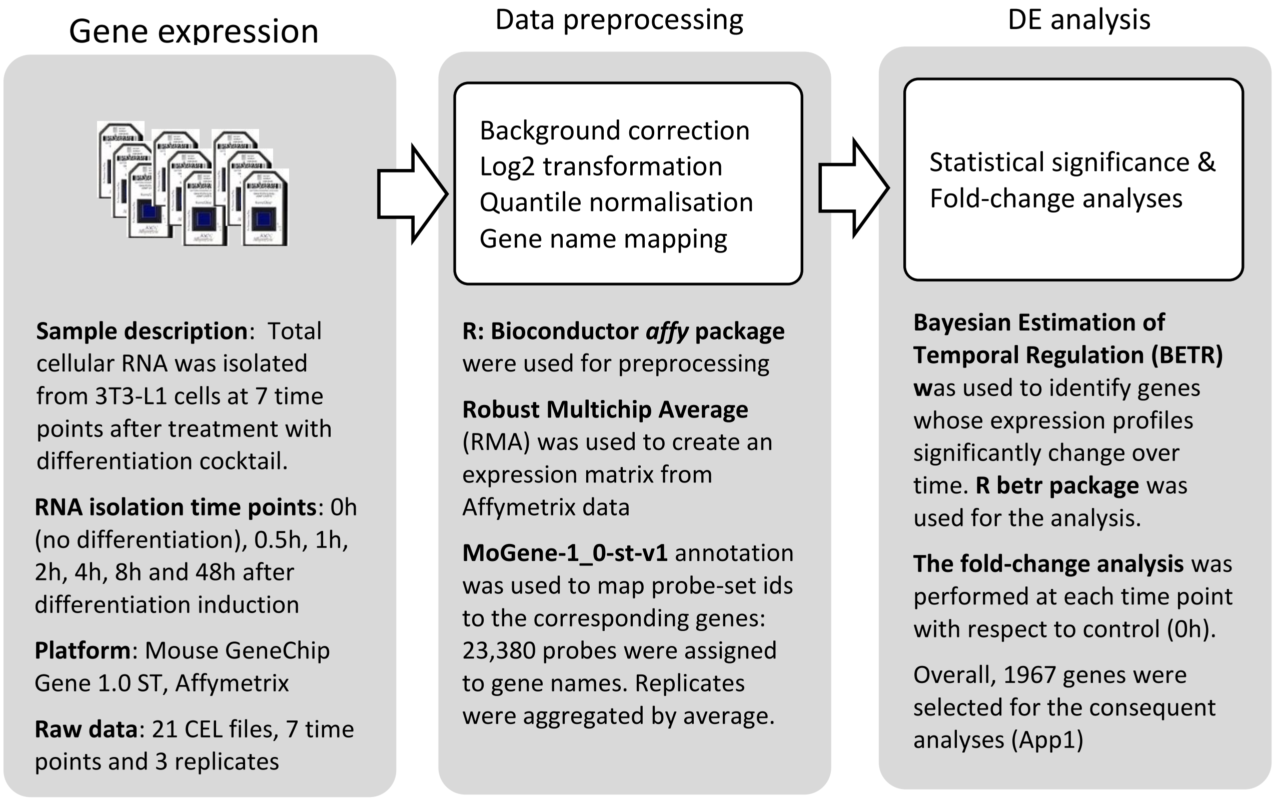

Figure A: The data preprocessing and differential expression (DE) analyses conducted for the adipogenesis time-course data (ref 17) used as a case study for Application 1.
